# Differential mechanisms of tolerance to extreme environmental conditions in tardigrades

**DOI:** 10.1038/s41598-019-51471-8

**Published:** 2019-10-17

**Authors:** Dido Carrero, José G. Pérez-Silva, Víctor Quesada, Carlos López-Otín

**Affiliations:** Departamento de Bioquímica y Biología Molecular, Facultad de Medicina, Instituto Universitario de Oncología del Principado de Asturias (IUOPA), Universidad de Oviedo, 33006 Oviedo, Spain

**Keywords:** Genome assembly algorithms, Zoology, Biodiversity

## Abstract

Tardigrades, also known as water bears, are small aquatic animals that inhabit marine, fresh water or limno-terrestrial environments. While all tardigrades require surrounding water to grow and reproduce, species living in limno-terrestrial environments (e.g. *Ramazzottius varieornatus*) are able to undergo almost complete dehydration by entering an arrested state known as anhydrobiosis, which allows them to tolerate ionic radiation, extreme temperatures and intense pressure. Previous studies based on comparison of the genomes of *R*. *varieornatus* and *Hypsibius dujardini* - a less tolerant tardigrade - have pointed to potential mechanisms that may partially contribute to their remarkable ability to resist extreme physical conditions. In this work, we have further annotated the genomes of both tardigrades using a guided approach in search for novel mechanisms underlying the extremotolerance of *R. varieornatus*. We have found specific amplifications of several genes, including *MRE11* and *XPC*, and numerous missense variants exclusive of *R. varieornatus* in *CHEK1*, *POLK*, *UNG* and *TERT*, all of them involved in important pathways for DNA repair and telomere maintenance. Taken collectively, these results point to genomic features that may contribute to the enhanced ability to resist extreme environmental conditions shown by *R. varieornatus*.

## Introduction

Tardigrades are small animals classically included in the clade Panarthropoda, together with Arthropoda and Onychophora. More than 1,200 species of tardigrades have been reported to inhabit all kinds of water environments. Even though they require surrounding water to grow and reproduce, limno-terrestrial tardigrades are well known for their remarkable capacity to endure extreme circumstances (such as dehydration, radiation, high and low temperature, high pressure, heavy metals and even outer-space conditions) when entering the anhydrobiotic state^1–6^. Nevertheless, some marine tardigrade species, such as *Echiniscoides sigismundi*, also present the ability to resist extreme dessication and intense gamma radiation^7,8^. Studies focused on survival and reproduction indicate that *R. varieornatus* presents a longer lifespan than *H. dujardini*^5^.

The study of the genomic sequence of one of the most stress-tolerant limno-terrestrial tardigrade species, *R. varieornatus*, has reported genomic alterations such as the expansion of several stress-related genes and the selective loss of peroxisomal oxidative and autophagy-related pathways, which can contribute to their tolerance to extreme environmental conditions^9^. Parallel studies have addressed the genome characterization of freshwater tardigrades, such as *H. dujardini*, which are among the least desiccation-resistant members of the phylum Tardigrada^10^, since they require previous conditioning to desiccation before entering anhydrobiosis. Such studies have also revealed various modifications in genes involved in macromolecule protection and stress signaling pathways that could contribute to the biological features exhibited by this tardigrade species, which lacks the extreme tolerance of *R. varieornatus*^11^. Other genomic comparative analyses have previously contributed to elucidate the mechanisms underlying aspects such as cancer resistance or longevity in different species^11–16^.

These genomic data have also revealed in *R. varieornatus* the presence of a novel tardigrade-unique protein called Dsup (damage suppressor) that suppresses X-ray-induced DNA damage and improves radiotolerance^9^. Nonetheless, recent studies found a Dsup homologue in *H. dujardini* that, despite its weak similarity with *R. varieornatus* Dsup, also presents nuclear localization and similar profiles in hydrophobicity and charge distribution along the protein^17^. This finding suggests that additional factors are involved in *R. varieornatus* extraordinary resistance to extreme conditions in comparison to *H. dujardini*, therefore encouraging the search for new hypotheses that explain the extremotolerance differences shown by these tardigrade species.

In this work, we have further explored the molecular mechanisms conferring extreme tolerance to limno-terrestrial tardigrades by comparing the genomes of *R. varieornatus* and *H. dujardini*, as well as that of a distant arthropod (*Drosophila melanogaster*). To this purpose, we have performed exhaustive manual annotation in these genomes of more than 250 genes involved in different DNA repair mechanisms. This comparative genomic analysis, together with the experimental validation of the identified alterations, has allowed us to detect specific gene amplifications and residue alterations in proteins involved in DNA repair pathways that may contribute to the enhanced tolerance to extreme environments exhibited by *R. varieornatus*.

## Methods

### Gene selection

Prior to genome annotation, we curated a list of more than 250 genes involved in oxygen homeostasis, stress response, telomere maintenance and DNA repair. Each gene was selected based on the experience of our laboratory in these fields^18–23^, and following a detailed revision of the available publications on each subject.

### Genomic analysis

We performed manual annotation of genomes *H. dujardini* (assembly 3.1, GCA_002082055.1) and *R. varieornatus* (assembly 4.0, GCA_001949185.1) using the BATI algorithm (Blast, Annotate, Tune, Iterate)^24^, that allows researchers to annotate the coordinates and intron/exon boundaries of genes in novel genomes from Tblastn results. This procedure also enables the user to identify novel homologues. In addition to each genome, the algorithm was fed reference sequences from *D. melanogaster* and automatically-annotated *H. dujardini* (obtained from Ensembl and NCBI databases). This supporting information contributes to generate homology-based alignments that are later interpreted and revised manually, thus allowing the researcher to apply the experience in defying genes and obtaining better and more precise genomic structures (especially in the case of the aforementioned exon/intron boundaries). Once the selected genes were properly annotated, we compared the resulting sequences of *R. varieornatus* and *H. dujardini* to those of human, chimpanzee (*Pan troglodytes*), mouse (*Mus musculus*), naked mole rat (*Heterocephalus glaber*), dog (*Canis lupus familiaris*), chicken (*Gallus gallus*), zebrafish (*Danio rerio*), Japanese rice fish (*Oryzias latipes*), coelacanth (*Latimeria chalumnae*), fruit fly *(D. melanogaster*) and roundworm (*Caenorhabditis elegans*) when available. This allowed the identification of gene expansions and losses, as well as residue changes specific of *R. varieornatus* and *H. dujardini*. In the alignment of TERT, we also included the HIV-1 RT sequence. In the alignment of POLK, we also included the prokaryotic species *Bdellovibrio bacteriovorus*, *Clostridium tetani*, *Escherichia coli*, *Mesorhizobium japonicum* and *Mycobacterium tuberculosis*. We evaluated the putative effects of these residue changes using data from NCBI Conserved Domains, UniProt and ClinVar databases.

### PCR analysis

To validate copy-number variations of genes of interest that we obtained through manual annotation, we performed PCR reactions with primer pairs that amplified a target region of the genomes of *R. varieornatus* and *H. dujardini* with different nucleotide sequences in each copy (Supplementary Table 1), and then examined the resulting electropherogram for evidence of both copies. *R. varieornatus* tardigrades were kindly provided by Dr. Takekazu Kunieda, University of Tokyo, Japan, while *H. dujardini* tardigrades were obtained from Sciento. Samples consisted of 50 tardigrades per species, which were snap frozen with liquid nitrogen. DNA was extracted using the QIAamp DNA Micro Kit (Qiagen). We tested the success of the PCR reactions by electrophoresis of the resulting products in a 1.5% agarose gel. Finally, the products were sequenced using the Sanger method and an ABI PRISM 3130xl Genetic Analyzer (Thermofisher). The results of the manual annotation and PCR analysis were also confirmed through RNA-Seq data from *H. dujardini* and *R. varieornatus* present into the NCBI Sequence Read Archive (SRA).

### Homology models

Homology models of selected proteins were performed with SWISS-MODEL^25^ and used to evaluate the potential function of the residues analysed in this manuscript. The sequences of CHK1 and POLK from *R. varieornatus* were modelled using structure 1jx4 and 3jvr as a template, respectively. Similarly, the sequence of UNG from *R. varieornatus* was modelled using structure 1q3f as a template. The resulting structure was aligned to structure 1ssp to study its putative mode of interaction with a DNA substrate. The results were inspected and rendered with DeepView v4.1.0. Electric potentials were calculated with DeepView using the Poisson-Boltzmann computation method. Figures were generated with PovRay (http://povray.org) and UCSF Chimera^26^.

## Results and Discussion

### Manual annotation of genes involved in DNA repair, stress response, telomere maintenance and oxygen homeostasis in tardigrades

To study the molecular mechanisms linked to the increased resistance to extreme environmental conditions shown by the tardigrade species *R. varieornatus* in comparison to *H. dujardini*, we analyzed a set of more than 250 genes involved in stress response, oxygen homeostasis, telomere maintenance and DNA repair (Table 1). Manual annotation of this gene set allowed us to find copy-number variations in genes related to DNA repair pathways, as well as to verify the previously described variations for both species of tardigrades. Interestingly, our analysis only revealed copy number variations between the two species of tardigrades in genes related to DNA repair mechanisms, particularly in genome maintenance during replication, double-strand break (DSB) repair, and nucleotide excision repair (NER) pathways (Table 2; Supplementary Table 2). However, no relevant copy number alterations were found in genes related to telomere maintenance, stress response or oxygen homeostasis when comparing the genomes of *R. varieornatus* and *H. dujardini*. Moreover, our analysis of DNA repair pathways in tardigrades and their comparison with reported data on human sequences led us to identify a series of residue changes that are exclusive of *R. varieornatus* and/or *H. dujardini* (Supplementary Table 3).

**Table 1:** List of genes analysed in this study.

ADGB	CCNH	EIF2AK4	FANCD2	GTF2H1	JUNB	NGB	POLM	REV3	TP53
ALKBH2	CDK7	EIF2S1	FANCE	GTF2H2	JUND	NHEJ1	POLN	RIF1	TPP1
ALKBH3	CETN2	EIF2S2	FANCF	GTF2H3	LIG1	NHP2	POLQ	RMI2	TREX1
APEX1	CHAF1A	EIF2S3	FANCG	GTF2H4	LIG3	NOP10	POT1	RNF168	TREX2
APEX2	CHEK1	EME1	FANCI	GTF2H5	LIG4	NTHL1	PRKDC	RNF4	TSC1
APOLD1	CHEK2	EME2	FANCL	H2AFX	MAD2L2	NUDT1	PROC	RNF8	TSC2
APTX	CLK2	ENDOV	FANCM	HBA1	MB	ODF1	PRPF19	RPA1	UBE2A
ARNTL	CLOCK	ENOX1	FEN1	HBB	MBD4	OGG1	PTGS1	RPA2	UBE2B
ATM	CRY1	ENOX2	FOS	HBZ	MDC1	PALB2	PTGS2	RPA3	UBE2N
ATR	CRY2	EPAS1	FOSB	HELQ	MGMT	PARP1	RAD1	RPA4	UBE2V2
ATRIP	CRYAA	ERCC1	FOSL1	HIF1A	MLH1	PARP2	RAD17	RRP1	UNG
BAD	CRYAB	ERCC2	FOSL2	HIF1AN	MLH3	PARP3	RAD18	SEM1	UVSSA
BAK1	CTC1	ERCC3	FOXO1	HIF3A	MMS19	PCNA	RAD23A	SETMAR	VHL
BCL2A1	CYGB	ERCC4	FOXO3	HLTF	MNAT1	PER1	RAD23B	SHPRH	VHLL
BCL2L1	DCLRE1A	ERCC5	FOXO4	HP	MPG	PER2	RAD50	SLX1A	WRN
BCL2L10	DCLRE1B	ERCC6	FOXO6	HSBP1	MPLKIP	PLAT	RAD51	SLX4	XAB2
BCL2L11	DCLRE1C	ERCC8	GADD45A	HSF1	MRE11	PLAU	RAD51B	SMUG1	XPA
BCL2L12	DDB1	ERN1	GADD45B	HSF2	MSH2	PLG	RAD51C	SPO11	XPC
BCL2L13	DDB2	ERN2	GADD45G	HSF3	MSH3	PMS1	RAD51D	SPRTN	XRCC1
BCL2L14	DKC1	EXO1	GAR1	HSF4	MSH4	PMS2	RAD52	STN1	XRCC2
BCL2L15	DMC1	F10	GEN1	HSF5	MSH5	PNKP	RAD54B	TDG	XRCC3
BCL2L2	Dsup	F11	GPX1	HSPA	MSH6	POLB	RAD54L	TDP1	XRCC4
BLM	DUT	F7	GPX2	HSPA12A	MUS81	POLD1	RAD9A	TDP2	XRCC5
BNIP2	EGLN1	FAAP20	GPX3	HSPA12B	MUTYH	POLE	RBBP8	TEN1	XRCC6
BOK	EGLN2	FAAP24	GPX4	HSPB	NABP2	POLG	RDM1	TERF1	ZFAND2A
BRCA1	EGLN3	FAN1	GPX5	HSPH1	NBN	POLH	RECQL	TERF2	ZFAND2B
BRCA2	EIF2AK1	FANCA	GPX6	HUS1	NEIL1	POLI	RECQL4	TERT	
BRIP1	EIF2AK2	FANCB	GPX7	HYOU1	NEIL2	POLK	RECQL5	TINF2	
CAT	EIF2AK3	FANCC	GPX8	JUN	NEIL3	POLL	REV1	TOPBP1

**Table 2:** Genes showing copy-number variations or residue changes in *R. varieornatus* in comparison to *H. dujardini*, classified into the main repair mechanisms that they are involved in.

Gene	Status in *R. varieornatus*	Status in *H. dujardini*	DNA repair mechanism
*CHEK1*	Residue change (p.F93Y)	No changes	DNA repair during replication, homologous recombination
*LIG4*	Amplification (two copies)	No changes	DNA repair during replication, non-homologous end joining
*XPC*	Amplification (two copies)	No changes	Nucleotide excision repair
*MRE11*	Amplification (four copies)	No changes	Non-homologous end joining, homologous recombination
*UNG*	Residue change (p.P177R)	No changes	Base excision repair
*RAD51*	Amplification (three copies)	No changes	Homologous recombination
*ERCC4*	Amplification (two copies)	No changes	Homologous recombination
*POLK*	Residue change (p.S132G)	No changes	Translesion synthesis
*REV1*	Residue change (p.A509S)	No changes	Translesion synthesis

In this study, we focused on the description of copy number variations and residue changes exclusive of the extreme tolerant *R. varieornatus* that lay in active sites or DNA binding sites, and involve genes important for homologous recombination, base excision repair, nucleotide excision repair, non-homologous end-joining, translesion synthesis, DNA repair during replication (Table 2), and for telomere dynamics.

### Telomere dynamics in *R. varieornatus* and *H. dujardini*

**Figure 1:**
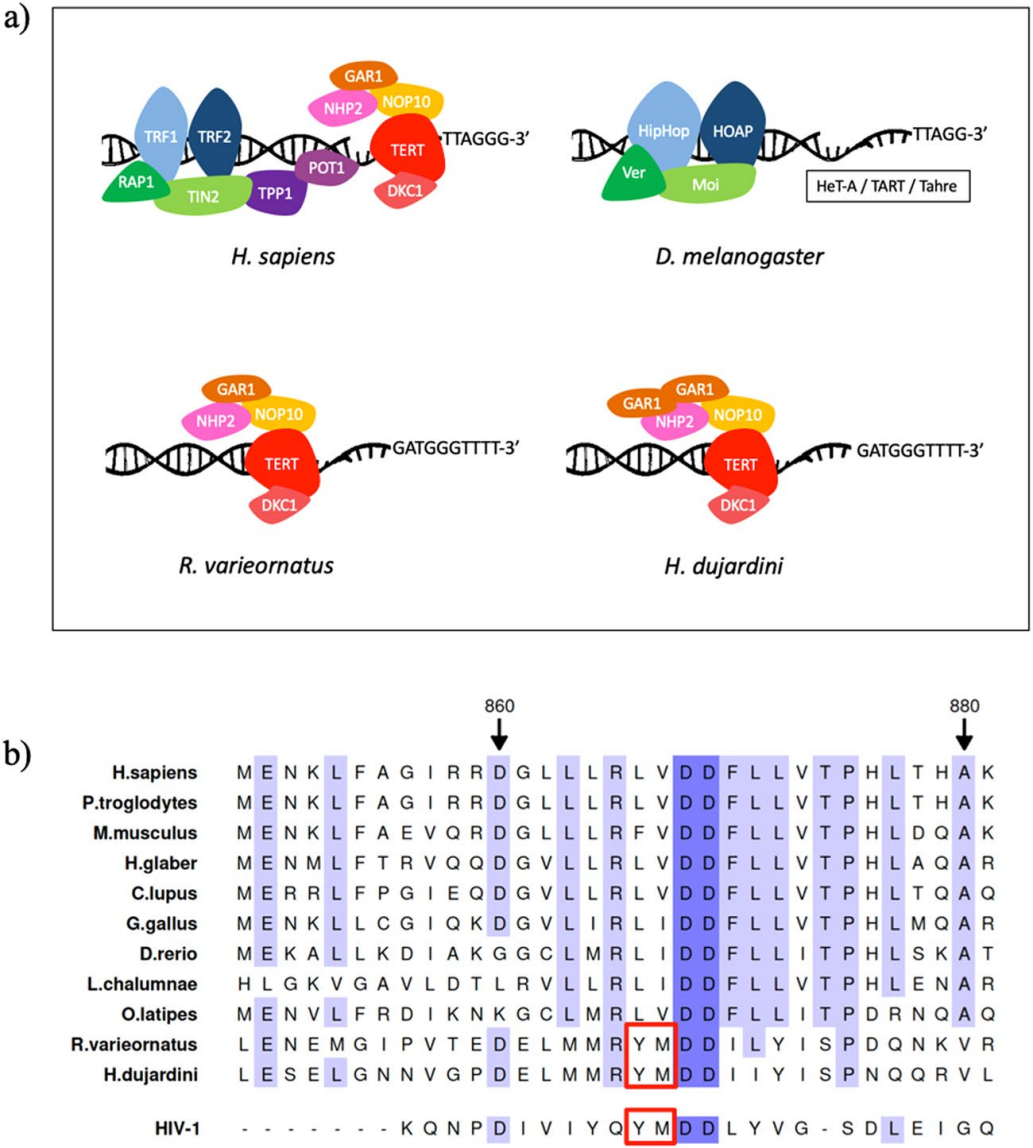
Telomere architecture in tardigrades compared to human and fruitfly. (**a**) Telomerase and telomere-capping complexes of human, fruitfly and tardigrades. Humans possess the shelterin complex (TRF1, TRF2, RAP1, TIN2, TPP1 and POT1), while *Drosophila* has the terminin complex (HipHop, HOAP, Ver and Moi), and tardigrades (*R. varieornatus* and *H. dujardini*) lack a telomere-capping complex. The telomerase complexes of humans and tardigrades are very similar, while in *Drosophila* telomeres replicate using a retrotransposon machinery composed of the elements HeT-A, TART and Tahre. (**b**) Partial amino acid sequence alignment of the TERT sequence in *R. varieornatus*, *H. dujardini* and other species of interest. Variants p.L866Y and p.V867M present in *R. varieornatus*, *H. dujardini* and HIV-1 reverse transcriptase are indicated with a red rectangle.

Telomeres have been widely studied in all Arthropoda, being their ancestral sequence (TTAGG)_n_ common to hexapods, crustaceans, myriapods, pycnogonids and most chelicerates, but not to spiders^27^. Nonetheless, such repeat sequence is absent in Tardigrada and Onychophora, which are closely related to Arthropoda. Thus, Onychophora present the vertebrate motif (TTAGGG)_n_, while tardigrades do not exhibit this telomere sequence either^27^. Further analysis of repeat sequences in the genome of *H. dujardini* revealed the presence of (GATGGGTTTT)_n_ repeats, which were exclusively found at 9 scaffold ends and are thought to correspond to telomeric sequences^11^ located in its 5 pairs of chromosomes^28^. Moreover, tardigrades and most arthropods lack the TERT motif CP, with the exceptions of hymenopterans and some centipedes^29^. This motif, together with the T motif, forms an extended pocket (T-CP pocket) on the surface of the protein implicated in RNA recognition and binding^30^. Remarkably, telomere elongation in *D. melanogaster* is carried out by three specialized retrotransposable elements (HeT-A, TART and Tahre)^31^, while no ortholog for the human gene *TERT* has been reported. In addition, fruit fly telomeres are capped by the complex terminin, functionally but not structurally analogous to shelterin, which includes the proteins HOAP, HipHop, Moi and Ver^32,33^ (Fig. 1a). These data indicate that telomere elongation and maintenance are carried out through different mechanisms in this species in contrast to other members of the Metazoa group.

In this work, we manually annotated several genes that encode proteins belonging to the telomerase, shelterin and CST complexes in tardigrades (Fig. 1a). Except for *TPP1*, none of the other components from the shelterin (*TERF1*, *TERF2*, *RAP1*, *POT1*, and *TINF2*) and CST (*CTC1*, *STN1* and *TEN1*) complexes were identified (Fig. 1a, CST complex not shown). Interestingly, we found in tardigrades a *bona fide TERT* ortholog, together with copies encoding all the elements of the telomerase complex, namely *NHP2*, *NOP10*, *DKC1* and *GAR1* (the latter being duplicated in *H. dujardini*) (Fig. 1a). Remarkably, two residue changes in TERT protein - p.L866Y and p.V867M - were found to be exclusively present in *H. dujardini* and *R. varieornatus* (Fig. 1b). Both residues are part of a tetrapeptide that includes a catalytically essential aspartate dyad (residues D868 and D869)^34^. These residues have been studied based on the previous discovery of the function of Y183 and M184, cognate amino acids to human TERT L866 and V867 in HIV-1 reverse transcriptase (Fig. 1b), which play important roles in processing, fidelity, enzymatic activity, dNTP utilization and nucleoside analogue inhibitor resistance^35^. These functional studies in human TERT have shown that the first variant alone (p.L866Y) results in a moderate reduction in telomerase activity, but produces no changes in repeat extension rate or in nucleotide incorporation fidelity^34^. The second variant (p.V867M) causes a 75% reduction in telomerase activity, 50% reduction in repeat extension rate, and 5.2-fold increase in nucleotide incorporation fidelity^34^. However, when both variants are present, they result in a slight reduction in telomerase activity and 13.5-fold increase in nucleotide incorporation fidelity^34^. This finding suggests that telomere dynamics in tardigrades may display reduced telomerase activity but also enhanced replication fidelity to prevent genomic instability caused by defects in telomere maintenance^20^.

### Alterations in genes involved in DNA repair and genome maintenance during replication in tardigrades

DNA ligation is essential for replication and repair, and genetic deficiencies in human DNA ligases have been associated with clinical syndromes characterized by radiation sensitivity and defects in DNA repair during replication through nonhomologous end joining (NHEJ)^36^. In mammals, this functional role is carried out by a protein family encoded by three genes (*LIG1*, *LIG3* and *LIG4*), all of them also present in *D. melanogaster*. While both tardigrade species seem to have one copy of *LIG1* and none of *LIG3*, we found two copies of *LIG4* in the genome of *R. varieornatus* (called *LIG4_1* and *LIG4_2*), while only one full copy and what could be one exon of another copy were detected in the genome of *H. dujardini*. The presence of this second *LIG4* copy in *H. dujardini* could not be verified by RNA-Seq nor Sanger sequencing due to the shortness of its contig (Supplementary Table 4), even though a putative expansion of *LIG4* in *H. dujardini* has been previously suggested^11^. Nevertheless, supporting data in this regard are not available in public repositories of genomic data^11^. Importantly, patients with null mutations in *LIG4* show increased sensitivity to ionizing radiation, as well as immunodeficiency, growth failure, and microcephaly^37^. In mice, Lig4 deficiency causes embryonic lethality due to a defective p53-dependent response to unrepaired DNA damage, as well as neuronal apoptosis and arrested lymphogenesis^38^. Moreover, mice with a hypomorphic mutation in *Lig4* show high levels of DNA DSBs during embryonic development and a deficient DSB repair response^39^. Accordingly, LIG4 mediates Wnt/β-catenin signaling activation during radiation-induced intestinal regeneration and blocking LIG4 sensitizes colorectal cancer cells to radiation^40^. Since the second copy of *H. dujardini* is not experimentally supported, it is plausible that the exclusive presence of two copies of *LIG4* in *R. varieornatus* might contribute to its enhanced resistance to DNA damage.

**Figure 2:**
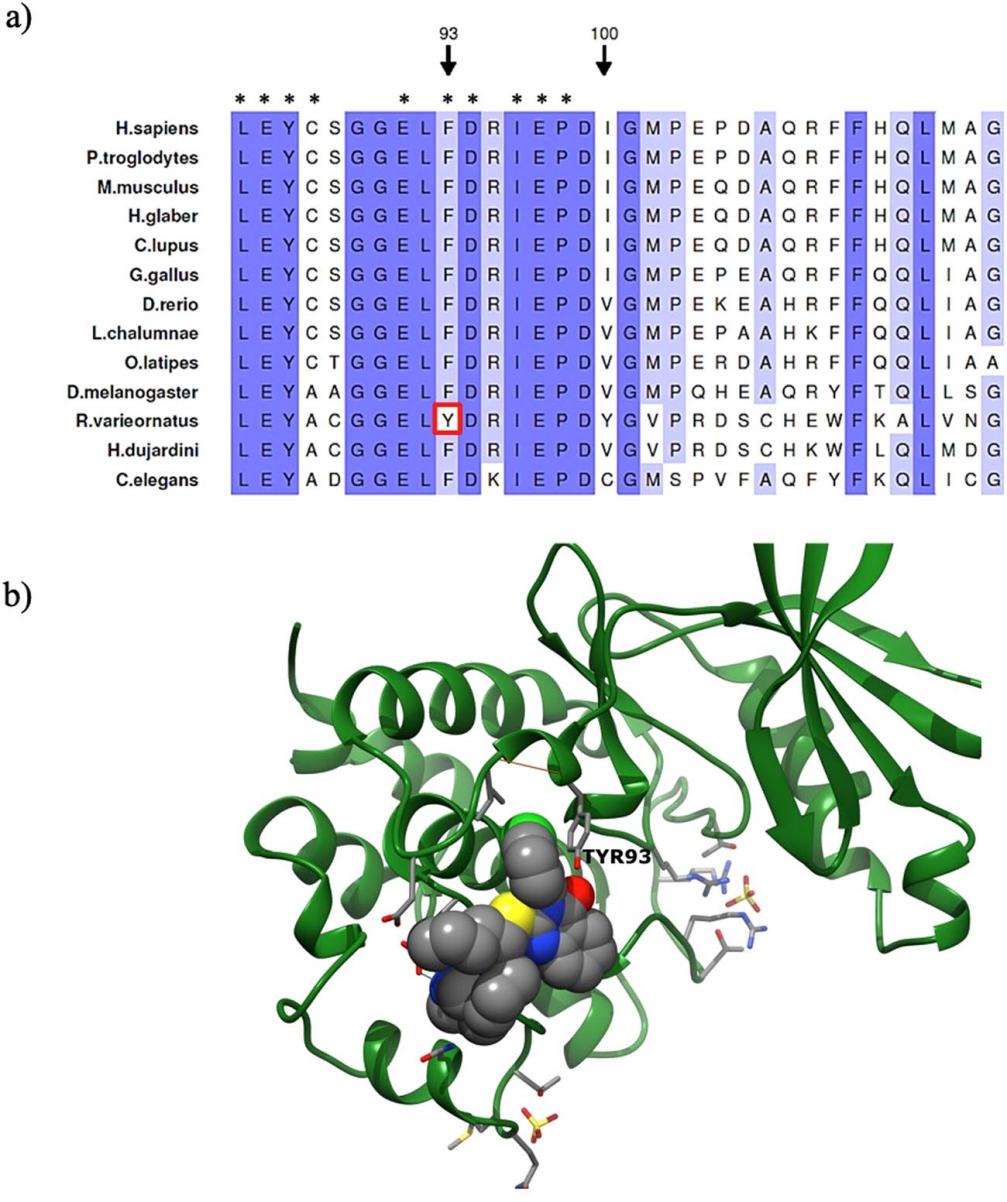
Comparative sequence analysis and homology modeling of CHK1 from *R. varieornatus*. (**a**) Partial amino acid sequence alignment of the CHK1 sequence in *R. varieornatus*, *H. dujardini* and other species of interest. Variant p.F93Y present in *R. varieornatus* is highlighted with a red rectangle. Important residues for its function are marked with *. (**b**) Representative image of the residue Y93 in the homology model of CHK1 from *R. varieornatus*. The homology model shows that the residue Y93, exclusive of *R. varieornatus*, that is defined in its wild-type form (F93) as an allosteric inhibitor binding site, is able to form an H-bond with the allosteric inhibitor that cannot be formed in its wild-type form (F93).

Moreover, we found several remarkable residue changes in *CHEK1* (Supplementary Table 3), which codes for the protein kinase CHK1 involved in DNA damage response (DDR), cell cycle arrest, and homologous recombination (HR)^41^. Among these *CHEK1* variants, we focused our attention on p.F93Y, exclusive of *R. varieornatus* (Fig. 2a), which affects an active site that functions as an allosteric inhibitor binding site and as a polypeptide substrate binding site^42^. To explore the putative functional relevance of this change, we generated a homology model of this protein in *R. varieornatus* (Fig. 2b). This model revealed that position 93 is located at the surface of a pocket in which allosteric inhibitors can be fitted, and showed the potential of the residue Y93 to form an H-bond with a synthetic allosteric inhibitor (Fig. 2b)^42^. This amino acidic change might influence the allosteric regulation of CHEK1 in *R. varieornatus* in comparison to *H. dujardini*. This regulatory mechanism may be important for its function, since CHK1 is involved in DNA damage response (DDR), cell cycle arrest, and homologous recombination (HR)^41^.

**Figure 3:**
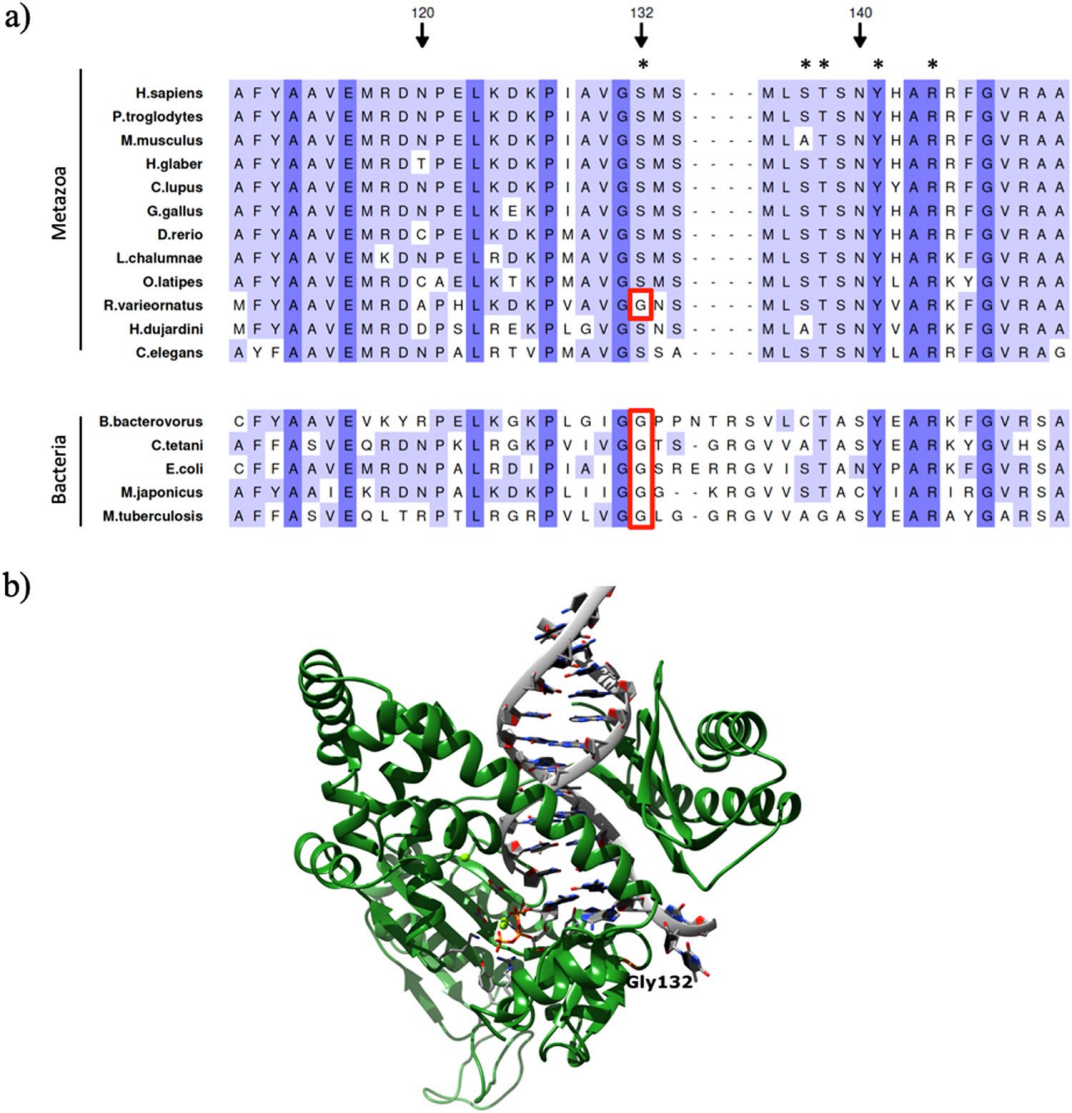
Comparative sequence analysis and homology modeling of POLK from *R. varieornatus*. (**a**) Partial amino acid sequence alignment of the POLK sequence in *R. varieornatus*, *H. dujardini* and other species of interest. Variant p.S132G present in *R. varieornatus* is indicated with a red rectangle. Important residues for its function are marked with *. (**b**) Representative image of the residue G132 in the homology model of POLK from *R. varieornatus*. The homology model shows that the residue G132, exclusive of *R. varieornatus*, that is defined in its wild-type form (S132) as DNA binding site, creates a more acute beta turn in the protein.

We also found an alteration (p.S132G) in the polymerase POLK exclusive of *R. varieornatus* (Fig. 3a), together with other residue changes shared with *H. dujardini* (Supplementary Table 3). The p.S132G variant affects a residue involved in DNA binding^43^. POLK is an error-prone DNA polymerase specifically involved in translesion synthesis during DNA replication, which preferentially incorporates adenine residues opposite to 8-oxoguanine lesions. These lesions frequently appear as a result of ionizing radiation, therefore producing missense mutations and frameshifts^43,44^. POLK appears to be absent in all arthropods. Its prokaryotic ortholog, DNA polymerase IV^45^, is also involved in repair of 8-oxoguanine lesions, but incorporates cytosine instead of adenine opposite to 8-oxoguanine with high efficiency, thus avoiding potential mutations^46^. Notably, prokaryotic DNA polymerase IV also presents glycine instead of serine in residue 132 (Fig. 3a), which suggests that the presence of glycine may contribute to incorporating the right nucleotide during repair of 8-oxoguanine lesions, resulting in higher fidelity and decreasing the occurrence of point mutations. The homology model of this protein in *R. varieornatus* suggests that, although the position 132 is not strictly close to the 8-oxoguanine lesion, it contributes to creating a more acute beta turn (Fig. 3b). Finally, REV1 - another protein involved in translesion synthesis^47^ - presents a variant exclusive of *R. varieornatus* affecting a DNA binding site (p.A509S)^48,49^. Additionally, *R. varieornatus* REV1 presents other changes in DNA binding sites that are also found in *H. dujardini* (Supplementary Table 3).

Finally, the gene *MGMT*, which encodes a methyltransferase involved in repairing the naturally occurring mutations O^6^-methylguanine and O^4^-methylthymine during replication^50^, is present in *H. dujardini* but the corresponding ortholog in *R. varieornatus* had not been previously identified in manual and automatic annotations. However, we could confirm the presence of *MGMT* when performing PCR on the genome of *R. varieornatus* using oligonucleotides based on the corresponding *MGMT* sequence of *H. dujardini* (Supplementary Table 4). Accordingly, its apparent absence in *R. varieornatus* genome is likely due to errors in the currently available genome assembly for this tardigrade.

### Expansion of genes involved in double-strand break repair in tardigrades

DSBs are particularly damaging alterations, since they can lead to chromosome rearrangements and losses. These genomic lesions can be repaired through three mechanisms: NHEJ, HR and microhomology-mediated end joining (MMEJ)^51^. We confirmed that the human *MRE11* ortholog, involved in NHEJ and HR^52^, is at least quadrupled in *R. varieornatus*, while *H. dujardini* displays one copy (Supplementary Table 4), as it has previously been reported^9,11^. The remarkable expansion of this gene may be responsible for an enhanced ability to repair DNA damage^53^. Moreover, knockdown of *MRE11* impaired DSB repair in HeLa and CNE2 cells^54^, and upregulation of this protein in cancer cells following ionizing radiation promoted DNA repair^54^. Altogether, these data suggest an important role of *MRE11* ortholog in *R. varieornatus* in promoting DNA repair after exposure to ionizing radiation.

We also confirmed the previous finding that the RAD51 protein family, involved in DSB repair through HR^55^, is expanded in *R. varieornatus*^9^. However, according to our data, we propose that one of the four copies annotated in this tardigrade’s genome by Hashimoto *et al*. actually corresponds to the *XRCC2* ortholog, as assessed by performing blast of these sequences (deposited in the NCBI database) against the human genome. Therefore, according to our annotation, the genome of *R. varieornatus* contains three copies of RAD51. We independently found the presence of the other three copies in both tardigrades. Expansion of the DNA repair endonuclease XPF (encoded by the gene *ERCC4*), also involved in HR^56^, was reported in *H. dujardini*, since five copies of this gene were found in its genome^11^. However, only three sequences out of these five could be found in the NCBI database, two of which belong to very small polypeptides (<100 aa); and only one is available at Ensembl Tardigrades^11^. In turn, manual annotation of this gene revealed two copies of *ERCC4* in this species (named *ERCC4_1* and *ERCC4_2*), while only one copy was found in *R. varieornatus*. This duplication could be verified by RNA-Seq, but not using Sanger sequencing due to the high similarity between both copies, and the presence of repetitive sequences (Supplementary Table 4). Finally, and similarly to the case of *MGMT*, one copy of the gene *XRCC3*, also involved in HR, could be found in the genome of *H. dujardini*. Although this gene seemed to be absent in the genome of *R. varieornatus*, we detected it by PCR using oligonucleotides designed for *H. dujardini* (Supplementary Table 4).

### Changes in genes related to base excision repair in *R. varieornatus*

**Figure 4:**
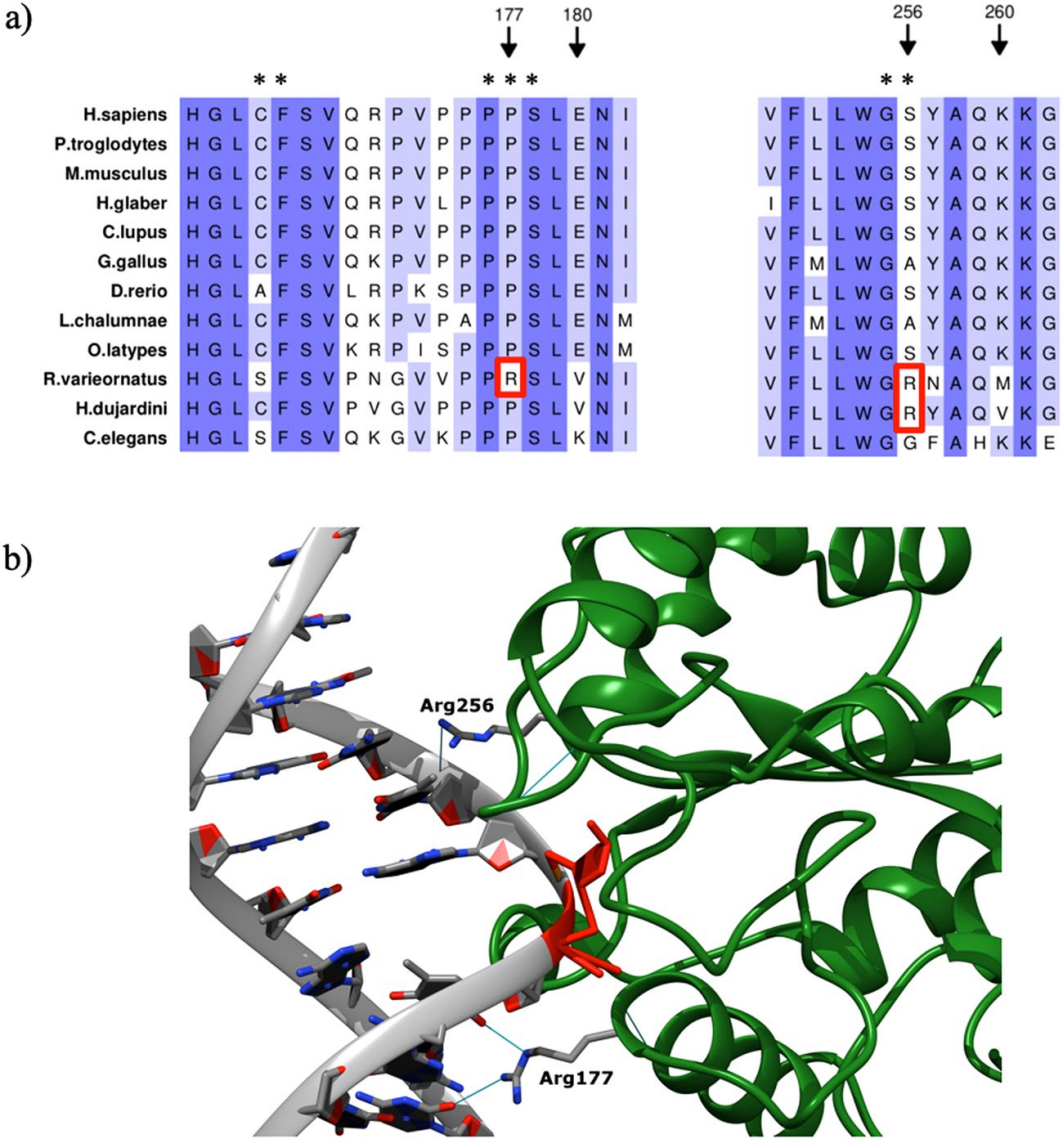
Comparative sequence analysis and homology modeling of *R. varieornatus* UNG bound to DNA. (**a**) Partial amino acid sequence alignment of the UNG sequence in *R. varieornatus*, *H. dujardini* and other species of interest. Variants p.P177R and p.S256R present in *R. varieornatus* are highlighted with a red rectangle. Important residues for its function are marked with *. (**b**) The enzyme is shown as a green ribbon. A DNA intermediate from structure 1ssp is shown in grey. The sugar from the substrate base is shown in red. UNG arginines 177 (specific of *R. varieornatus*) and 256 (specific of tardigrades) are labelled. Putative interactions involving R177 or R256 are shown as blue lines.

Among all genes involved in base excision repair (BER) analysed in *R. varieornatus* and *H. dujardini*, we found a variant in an active site and UGI (uracil-DNA glycosylase inhibitor protein) interface site (p.P177R) in the protein encoded by *UNG* that is exclusive of *R. varieornatus*^57^ (Fig. 4a). This protein is a DNA glycosylase that excises uracil residues from DNA when misincorporation of uracil occurs during DNA replication or due to deamination of cytosine^58^. The model predicts that Arg 177 fits the minor groove of the DNA molecule, very close to the everted base (Fig. 4b). This mode of interaction has been described previously in the context of the nucleosome, and it was found to be independent of the DNA sequence^59^, which suggests that this variant might contribute to the association of UNG to substrate DNA. In this regard, another tardigrade-specific arginine at position 256 (Fig. 4a) interacts with a phosphate group at the other side of the everted base. However, given the proximity of Arg177 to the substrate base, this model cannot rule out the possibility that this residue might also play a role in base eversion, as proposed in similar contexts for other enzymes^60^.

### Nucleotide excision repair in *R. varieornatus*

Oxidative DNA damage is considered as a leading cause of both neurodegeneration and cancer development as illustrated by syndromes that result from NER defects, such as Xeroderma pigmentosum (XP) and Cockayne syndrome (CS)^61,62^. Among all the genes involved in NER, *XPC* appears to be duplicated in *R. varieornatus* (with copies we have named *XPC_1* and *XPC_2*) but not in *H. dujardini* (Supplementary Table 4). This protein is involved in repair of damage caused by UV light, since mutations in the gene encoding this protein in humans lead to XP^61^, and *Xpc* knockout mice show an increased susceptibility to UVB induced squamous cell carcinomas^63^. Therefore, this duplication in the *XPC* ortholog in *R. varieornatus* may also contribute to the enhanced tolerance to radiation in this species by improving its NER response pathway.

### Summary

In this manuscript, we describe several gene expansions of pivotal elements in DNA repair pathways observed in the genomes of *R. varieornatus* and *H. dujardini* through manual annotation, including previously described expansions, such as *XPC*, *LIG4*, *ERCC4* and *MRE11*^9,11^. Manual genomic comparative analyses also revealed residue changes in key elements in DNA repair pathways that in the corresponding human orthologs are known to cause an effect in the function of the protein (Supplementary Table 3), among which we highlight the ones exclusively found in *R. varieornatus* in the genes *TERT*, *CHEK1*, *POLK* and *UNG*. However, considering the phylogenetic distance between tardigrades and humans, in most cases it is difficult to define the consequences of such variants in tardigrade proteins, and further experimental work is required to raise definitive conclusions in this regard. Nonetheless, these findings show that combining both manual and automatic annotation approaches is an advantageous strategy to better determinate the precise number of gene copies and to find residue changes when analyzing a genome *de novo*.

In short, all the changes we observed in *R. varieornatus* suggest an enhanced ability to maintain genomic stability, which may explain its resistance to extreme conditions, as well as its longer lifespan in comparison to *H. dujardini*. Additionally, the recent finding of a Dsup homologue in *H. dujardini*^17^ reinforces our proposal that specific features in DNA repair genes are important elements in the extraordinary resistance shown by this limno-terrestrial tardigrade species.

## Supplementary information

Supplementary information

## Data Availability

The manually annotated dataset of genes and proteins generated and analyzed during the current study supporting the conclusions of this article are available in a public repository in GitHub(https://github.com/EreboPSilva/rvar.hduj.prots).
