# Supplementary material for: Differential mechanisms of tolerance to extreme environmental conditions in tardigrades
Source: Sci Rep. 2019 Oct 17;9:14938. doi: 10.1038/s41598-019-51471-8 (PMC6797769; doi:10.1038/s41598-019-51471-8)
Supplement: Supplementary file 1 — Supplementary information [file 41598_2019_51471_MOESM1_ESM.pdf]

Differential mechanisms of tolerance to extreme environmental conditions in tardigrades

Dido Carrero, José G. Pérez-Silva, Víctor Quesada & Carlos López-Otín[1*]

Supplementary Table 1. Primer pairs used to identify copy number variations in *R. varieornatus* and *H. dujardini*.

**Gene**	***R. varieronatus***		***H. dujardini***	
	Fw primer	***Rv primer***	***Fw primer***	***Rv primer***
LIG4_1	CGTCAGTGTACAAGCCCAAC	TCGGCAAAAGGACAAGAACT	GTGTATCCGCTACCGACCTT	ACCGTTTACAAGCCCAACAA
LIG4_2	CGACAAGTCCTTCATCGTCA	CCGCAGTCTTCTGCTTCTTT	CGACAAGTCCTTCATCGTCA	CCGCAGTCTTCTGCTTCTTT
MGMT	GTAATTGCCCACGATTCCAG	CCGGTGCTTTTATACCACAGA	GTAATTGCCCACGATTCCAG	CCGGTGCTTTTATACCACAGA
MRE11_1	TCGAAGTTCTTGACGGAAGC	ACTTGGGCGAACTGAACGAGA	CGCCTGAATAATCTCCTTCG	TCGGGATGATGTGGAGAAAT
MRE11_2	TCGAAGTTCTTGACGGAAGC	ACTTGAACAGACTCAGCGAGA	TCGAAGTTCTTGACGGAAGC	ACTTGAACAGACTCAGCGAGA
MRE11_3	GGTCGAATGGAGCGTATCAT	GGCTTGATTTCCTTCGACAC	GGTCGAATGGAGCGTATCAT	GGCTTGATTTCCTTCGACAC
MRE11_4	TGCTCCGTTCCTACTGCATG	GCCGTACGTGTGGAGAAGAT	TGCTCCGTTCCTACTGCATG	GCCGTACGTGTGGAGAAGAT
XRCC3	CTGTCCGTTGCTGGATAAGC	AGTTAGCGCAAGGTTTTTGC	CTGTCCGTTGCTGGATAAGC	AGTTAGCGCAAGGTTTTTGC
ERCC4	CATCTGTTGCGGATCCATTA	TTGAAGTGCGTTGGTTCATC	AAGAACAGCGCTACCTCACC	ACGCATATCGACGATGACAC
XPC_1	GACAAAGCGATCCACGAGAT	CGCAACCAGTTCTCCCTAGT	CCTCCTTATACGGCTGCTCA	GGTGCAGGAGTTCAAAGGAC
XPC_2	GCGATTCGAGAACTCTTGCT	CTTCAGCCAGTTTTCCCTTG	GCGATTCGAGAACTCTTGCT	CTTCAGCCAGTTTTCCCTTG

Supplementary Table 2. Number of copies of genes that show amplifications or deletions in any of the species analyzed (human, fruitfly, and both tardigrade species).

Gene	*H. sapiens*	*D. melanogaster*	*R. varieornatus*	*H. dujardini*
ADGB	1	0	1	1
ALKBH2	1	0	0	0
ALKBH3	1	0	0	0
APEX1	1	1	0	0
APEX2	1	0	1	1
APOLD1	1	0	0	0
APTX	1	0	2	2
ATM	1	1	0	0
ATRIP	1	1	0	0
BAD	1	0	0	0
BAK1	1	0	0	0
BCL2A1	1	0	0	0
BCL2L1	1	0	0	0
BCL2L10	1	0	0	0
BCL2L11	1	0	0	0
BCL2L12	1	0	0	0
BCL2L13	1	0	0	0
BCL2L14	1	0	0	0
BCL2L15	1	0	0	0
BCL2L2	1	0	0	0
BOK	1	1	0	0
BRCA1	1	0	0	0
BRCA2	1	1	0	0
BRIP1	1	0	1	1
CAT	1	0	2	2
CDK7	1	1	2	2
CLOCK	1	1	0	0
CRY1	1	1	0	0
CRY2	1	1	0	0
CRYAA	1	0	0	0
CRYAB	1	0	0	0
CTC1	1	0	0	0
CYGB	1	0	0	0
DCLRE1B	1	0	0	0

DCLRE1C	1	0	1	1
DDB1	1	1	2	2
DDB2	1	0	0	0
DMC1	1	0	0	0
Dsup	0	0	1	1
EGLN1	1	1	0	0
EGLN2	1	0	0	0
EGLN3	1	0	0	0
EIF2AK1	1	0	1	1
EIF2AK2	1	0	0	0
EME2	1	0	0	0
ENDOV	1	0	1	1
ENOX1	1	1	0	0
ENOX2	1	1	0	0
EPAS1	1	0	0	0
ERCC4	1	1	1	2
ERCC5	1	1	1	2*
ERCC6	1	0	1	1
ERCC8	1	0	1	1
ERN2	1	0	0	0
F10	1	0	0	0
F11	1	0	0	0
F7	1	0	0	0
FAAP20	1	0	0	0
FAAP24	1	0	0	0
FAN1	1	0	1	1
FANCA	1	0	0	0
FANCB	1	0	0	0
FANCC	1	0	0	0
FANCD2	1	1	0	0
FANCE	1	0	0	0
FANCF	1	0	0	0
FANCG	1	0	0	0
FANCI	1	1	0	0
FANCL	1	1	0	0
FOS	1	0	0	0
FOSB	1	0	0	0
FOSL1	1	0	0	0

FOSL2	1	0	0	0
FOXO3	1	0	0	0
FOXO4	1	0	0	0
FOXO6	1	0	0	0
GADD45A	1	1	0	0
GADD45B	1	0	0	0
GADD45G	1	0	0	0
GAR1	1	0	1	2
GEN1	1	1	0	0
GPX1	1	0	0	0
GPX2	1	0	0	0
GPX3	1	0	0	0
GPX5	1	0	0	0
GPX6	1	0	0	0
GPX7	1	0	0	0
GPX8	1	0	0	0
HBA1	1	0	0	0
HBB	1	0	0	0
HBZ	1	0	0	0
HELQ	1	1	0	0
HIF1A	1	0	0	0
HIF1AN	1	0	0	0
HIF3A	1	0	0	0
HLTF	1	0	0	0
HP	1	0	0	0
HSF2	1	0	0	0
HSF3	1	0	0	0
HSF4	1	0	0	0
HSF5	1	0	0	0
HSPA12A	1	0	0	0
HSPA12B	1	0	0	0
JUNB	1	0	0	0
JUND	1	0	0	0
LIG3	1	1	0	0
LIG4	1	1	2	1
MB	1	0	0	0
MBD4	1	0	0	0
MDC1	1	0	0	0

MGMT	1	1	1^	1
MLH3	1	0	1	1
MPG	1	0	1	1
MPLKIP	1	0	0	0
MRE11	1	1	4	1
MSH3	1	0	0	0
MSH4	1	0	1	1
MSH5	1	0	1	1
MUTYH	1	1	0	0
NBN	1	1	0	0
NEIL1	1	0	1	1
NEIL2	1	0	0	0
NEIL3	1	0	0	0
NGB	1	0	0	0
NHEJ1	1	0	0	0
NTHL1	1	1	0	0
NUDT1	1	0	0	0
ODF1	1	0	0	0
PALB2	1	0	0	0
PARP2	1	0	1	1
PARP3	1	0	1	1
PCNA	1	2	2	2
PER1	1	1	0	0
PER2	1	0	0	0
PLAT	1	0	1	1
PLAU	1	0	0	0
PLG	1	0	0	0
PMS1	1	0	0	0
POLB	1	0	1	1
POLI	1	1	0	0
POLK	1	0	1	1
POLL	1	0	3	2
POLM	1	0	0	0
POLN	1	0	0	0
POLQ	1	1	2	2
POT1	1	0	0	0
PROC	1	0	0	0
PTGS1	1	0	0	0

PTGS2	1	0	0	0
RAD18	1	0	0	0
RAD51B	1	0	1	1
RAD51C	1	1	0	0
RAD52	1	0	0	0
RAD54B	1	0	0	0
RAD9A	1	1	0	0
RBBP8	1	0	0	0
RDM1	1	0	0	0
RECQL4	1	0	0	0
REV1	1	1	1	2*
RIF1	1	0	0	0
RMI2	1	0	0	0
RNF168	1	0	0	0
RNF4	1	0	0	0
RNF8	1	0	0	0
RPA3	1	1	0	0
RPA4	1	0	0	0
SEM1	1	0	0	0
SETMAR	1	1	0	0
SLX4	1	1	0	0
SMUG1	1	1	0	0
STN1	1	0	0	0
TDP2	1	0	1	1
TEN1	1	0	0	0
TERF1	1	0	0	0
TERF2	1	0	0	0
TERT	1	0	1	1
TINF2	1	0	0	0
TPP1	1	1	2	2
TREX1	1	0	0	0
TREX2	1	0	0	0
TSC1	1	1	0	0
TSC2	1	1	0	0
UBE2A	1	1	2	2
UBE2B	1	0	1	1
UNG	1	0	1	1
UVSSA	1	0	1	1

VHL	1	0	0	0
VHLL	1	0	0	0
WRN	1	1	0	0
XPC	1	1	2	1
XRCC3	1	1	1^	1
XRCC4	1	0	0	0
ZFAND2A	1	1	0	0
ZFAND2B	1	0	1	1

* duplicated genes in which the second copy presents several frameshifts and is likely not to be functional. ^ genes absent in the assembly but found by PCR using oligonucleotides designed for the other species.

Supplementary Table 3. Residue changes of interest in tardigrade proteins compared to the corresponding human proteins.

Gene	Variant	Importance in humans	Species
ATR	L2303M	ATP binding site	R.var
	L2303V	ATP binding site	H.duj
	F2496Y	Activation loop	H.duj
BNIP2	L211F	Phospholipid binding pocket	R.var, H.duj
	R238E, I253F	Phospholipid binding pocket	R.var
	R238Q, I253L	Phospholipid binding pocket	H.duj
BRIP1	V611I	Hereditary breast cancer, inherited cancer-predisposing syndrome	R.var
CDK7	Multiple variants	ATP binding site, active site	H.duj
CHEK1	F93Y	Allosteric inhibitor binding site, active site, polypeptide substrate binding site	R.var
	L164I	Activation loop	R.var, H.duj
	P172S	Activation loop, polypeptide substrate binding site, active site	R.var, H.duj
	L206Q	Allosteric substrate binding site	H.duj
CHEK2	M381L	Activation loop	H.duj
CLK2	F251Y	Active site, polypeptide binding substrate site	R.var, H.duj
	I340T, S342T	Active site, polypeptide binding substrate site, activation loop	R.var, H.duj
DKC1	F36V	Dyskeratosis congénita X-linked (DKCX) [64]	R.var, H.duj
DUT	E210I, K212N	Trimer interface	R.var
	E210K, K212S	Trimer interface	H.duj
EIF2AK1	L212F	Dimer interface	R.var, H.duj
	Q385E, G528A	Active site, ATP binding site	R.var, H.duj
	T488S	Phosphothreonine	R.var, H.duj
	C466R, T488S	Activation loop	R.var, H.duj
EIF2AK3	V193I	Homodimer interface	R.var
	G197A, S217A	Homodimer interface	H.duj
	G954A	Activation loop	R.var, H.duj
EIF2AK4	L906M	eIF2 alpha binding site, activation loop, active site	H.duj
ENDOV	F95Y	Active site	R.var
ERN1	C109Q, D123A, G157P	Homodimer interface	R.var, H.duj
	E582D	Dimer interface	R.var, H.duj
	K908R, H909S	Dimer interface	R.var
FOXO1	Y196F	DNA binding site	R.var

	S256T	Phosphoserine	R.var, H.duj
GTF2H1	P57A, Q97M	Peptide binding site	R.var
	Q97T	Peptide binding site	H.duj
GTF2H2	S144T	Partial metal ion-dependent adhesion site	H.duj
HYOU1	G41S	Nucleotide binding site	R.var, H.duj
	S42N	Nucleotide binding site	R.var
	N515L	N-linked asparagine	R.var, H.duj
JUN	K226P	Glycyl lysine isopeptide (Lys-Gly)	R.var, H.duj
	S267A	DNA binding site	R.var, H.duj
	K271M	N6-acetyllysine	R.var, H.duj
	L274I	DNA binding site, dimer interface	R.var
	A295T	Dimer interface	H.duj
	L301T	Dimer interface	R.var
MLH1	R182K	HNPCC2 [65]	R.var, H.duj
MPG	S219G	DNA binding site	R.var, H.duj
	K220R	DNA binding site	H.duj
MSH5	G644R, T647A	ABC transporter signature motif	R.var, H.duj
NABP2	V23I, Y74F	Generic binding surface I	R.var, H.duj
	V43I	Generic binding surface I	R.var
	I67V	Generic binding surface II	H.duj
NEIL1	P5A	H2TH interface	R.var, H.duj
	H8A	H2TH interface	R.var
	R78G	Putative DNA binding site	R.var, H.duj
OGG1	Q315H	Oxoguanine	R.var, H.duj
PARP1	K600T	N6-acetyllysine	H.duj
PARP3	C183S, L192M, T259S	Parp regulatory domain	H.duj
	E231T, M285D	Parp regulatory domain	R.var
	M209L, N264D, K283F, V288T	Parp regulatory domain	R.var, H.duj
	A290G	Parp regulatory domain, inhibitor contact residues	R.var, H.duj
PLAT	D507T, S532V	Substrate binding site	R.var, H.duj
	S513G	Active site	R.var, H.duj
POLB	K61E	Glycyl-lysine isopeptide	R.var
	K61Q	Glycyl-lysine isopeptide	H.duj
	K72Q	Schiff base intermediate with DNA, N6-acetyllysine, abolishes DNA lyase activity. No effect on DNA polymerase activity [66]	R.var
	K72E	Schiff base intermediate with DNA, N6-acetyllysine	H.duj

	K81Q	Glycyl-lysine isopeptide	H.duj
	R83K	Omega-N-methylarginine. Mutation abolishes methylation by PRMT6 and impairs polymerase activity when associated with K-152 [67]	H.duj, R.var (R83E)
	T101S	Sodium; via carbonyl oxygen, active site, primer binding site	R.var
	T101A	Sodium; via carbonyl oxygen, active site, primer binding site	H.duj
	P108I, A110R, K113E, H135S	Active site, primer binding site	R.var
	P108F, A110H, K113H, H135A	Active site, primer binding site	H.duj
	R149P, S180A, A185D, S187T, S188I	Active site, NTP binding site	R.var, H.duj
	D190E, D192V	Magnesium 1 y 2	R.var, H.duj
	G190L, K234R, N279I	Active site, NDP binding site, primer binding site	R.var
	G190T, K234G, N279V	Active site, NDP binding site, primer binding site	H.duj
	D191E, D193V	Active site, NDP binding site, metal binding site, primer binding site	R.var, H.duj
	M236K, F272W, S275N	Active site, NDP binding site, primer binding site	R.var, H.duj
	R254Y	Active site, NTP binding site	R.var
	R254L	Active site, NTP binding site	H.duj
	D256A	Magnesium 1 y 2, active site, NDP binding site, metal binding site, primer binding site	R.var
	D256S	Magnesium 1 y 2, active site, NDP binding site, metal binding site, primer binding site	H.duj
POLK	A110M	Active site	R.var, H.duj
	S132G	DNA binding site	R.var
	D445S	DNA binding site	R.var, H.duj
PRKDC	P3956A	Activation loop	H.duj
PRPF19	K179L	N6-acetyllysine	R.var
	K179R	N6-acetyllysine	H.duj
	K244G	N6-acetyllysine	R.var, H.duj
	S333A, D371N, W377Y, D420H	Structural tetrad	R.var
	G366A, D371R, D378E, T409S	Structural tetrad	H.duj
RECQL	K514Q	N6-acetyllysine	R.var, H.duj

REV1	I361L, H774C	DNA binding site	R.var, H.duj
	A509S	DNA binding site, active site	R.var
RPA1	K259N, K267N, K410L	Glycyl-lysine isopeptide	R.var
	K220E, K259G, K267D, K410S	Glycyl-lysine isopeptide	H.duj
RPA2	D103E, R133K	Generic binding surface I	R.var, H.duj
SLX1A	V16C	GIY/YIG motif-motif A	R.var, H.duj
TDG	I139F, K232L, F252L, A274P	Active site	R.var, H.duj
	G156A	Active site	R.var
	F164Y, H287R, I290E	SUMO-interface	R.var
	F164H, H287K, I290Q	SUMO-interface	H.duj
	V286L	SUMO-interface	R.var, H.duj
TERT	L841F	Pulmonary fibrosis, and/or bone marrow failure, telomere-related, 1 (PFBMFT1)[68]	R.var
	L866Y	Moderate reduction in telomerase activity, no change in repeat extension rate nor on nucleotide incorporation fidelity. Little further reduction in activity but 13.5-fold increase in nucleotide incorporation fidelity; when associated with M-867 [34]	R.var, H.duj
	V867M	About 75% reduction in telomerase activity, about 50% reduction in repeat extension rate and 5.2-fold increase in nucleotide incorporation fidelity. Small further reduction in activity and 13.5-fold increase in nucleotide incorporation fidelity; when associated with Y-866 [34]	R.var, H.duj
TOPBP1	G132A	Dimer interface	R.var
	S270C, Y1333F	BRCT sequence motif	H.duj
TP53	H179S	Dimerization site, Zinc binding site	R.var
	C277A	DNA binding site	R.var
UBE2B	K75R	Ub thioester intermediate interaction residues	R.var, H.duj
	T99S	E3 interaction residues	R.var, H.duj
UBE2V2	K85R	Ub thioester intermediate interaction residue	H.duj
UNG	P177R	Active site, UGI interface	R.var
	K295Q	N6-acetyllysine	R.var
	K295R	N6-acetyllysine	H.duj
XRCC2	T55S	ATP binding site, walker A motif	R.var, H.duj
	T83I	ATP binding site	R.var, H.duj

	L146I	Walker B motif	R.var, H.duj
XRCC3	G112A	Walker A motif	H.duj
XRCC5	S258K	Heterodimer interface, phosphoserine	R.var, H.duj
	K274Q, G313A, V375T, D455G, L463V, P483R	Heterodimer interface	R.var, H.duj
	E292T, T293Q, I311V	Heterodimer interface	R.var
	E292I, T293S, I311G	Heterodimer interface	H.duj

R.var: *R. varieornatus*; H.duj: *H. dujardini*.

Supplementary Table 4. Validation of candidate genes to explain *R. varieornatus* extremotolerance.

**Gene**	***R. varieronatus***	***H. dujardini***
LIG4_1	Validated through RNA-Seq and Sanger sequencing	Validated through RNA-Seq and Sanger sequencing
LIG4_2	Validated through RNA-Seq and Sanger sequencing	Could not be validated due to shortness of contig, not found by PCR using oligonucleotides from *R. var*
MGMT	Two adjacent genes in *H. duj* are present, found by PCR using oligonucleotides for *H. duj*	Validated through RNA-Seq and Sanger sequencing
MRE11_1	Validated through RNA-Seq and Sanger sequencing	Validated through RNA-Seq and Sanger sequencing
MRE11_2	Validated through RNA-Seq and Sanger sequencing	Not found by PCR using oligonucleotides from *R. var*
MRE11_3	Validated through RNA-Seq and Sanger sequencing	Not found by PCR using oligonucleotides from *R. var*
MRE11_4	Validated through RNA-Seq and Sanger sequencing	Not found by PCR using oligonucleotides from *R. var*
ERCC4_1	Validated through RNA-Seq and Sanger sequencing	Validated through RNA-Seq and Sanger sequencing
ERCC4_2		Validated through RNA-Seq, could not be validated through Sanger sequencing due to high similarity between copies and presence of repetitive sequences

XRCC3	One adjacent gene in *H. duj* is present, contig terminates on the other end, found by PCR using oligonucleotides for *H. duj*	Validated through RNA-Seq and Sanger sequencing
XPC_1	Validated through RNA-Seq and Sanger sequencing	Validated through RNA-Seq and Sanger sequencing
XPC_2	Validated through RNA-Seq and Sanger sequencing	Not found by PCR using oligonucleotides from *R. var*

R.var: *R. varieornatus*; H.duj: *H. dujardini*.

Supplementary references

64 Heiss, N. S. *et al.* X-linked dyskeratosis congenita is caused by mutations in a highly conserved gene with putative nucleolar functions. *Nat Genet* **19**, 32-38, doi:10.1038/ng0598-32 (1998).

65 Taylor, C. F., Charlton, R. S., Burn, J., Sheridan, E. & Taylor, G. R. Genomic deletions in MSH2 or MLH1 are a frequent cause of hereditary non-polyposis colorectal cancer: identification of novel and recurrent deletions by MLPA. *Hum Mutat* **22**, 428-433, doi:10.1002/humu.10291 (2003).

66 Matsumoto, Y., Kim, K., Katz, D. S. & Feng, J. A. Catalytic center of DNA polymerase beta for excision of deoxyribose phosphate groups. *Biochemistry* **37**, 6456-6464, doi:10.1021/bi9727545 (1998).

67 El-Andaloussi, N. *et al.* Arginine methylation regulates DNA polymerase beta. *Mol Cell* **22**, 51-62, doi:10.1016/j.molcel.2006.02.013 (2006).

68 Tsakiri, K. D. *et al.* Adult-onset pulmonary fibrosis caused by mutations in telomerase. *Proc Natl Acad Sci U S A* **104**, 7552-7557, doi:10.1073/pnas.0701009104 (2007).

69 Drosopoulos, W. C. & Prasad, V. R. The active site residue Valine 867 in human telomerase reverse transcriptase influences nucleotide incorporation and fidelity. *Nucleic Acids Res* **35**, 1155-1168, doi:10.1093/nar/gkm002 (2007).
